# The “Counseling+” Roles of the Speech-Language Pathologist Serving Older Adults With Mild Cognitive Impairment and Dementia From Alzheimer’s Disease

**DOI:** 10.1044/2021_persp-20-00295

**Published:** 2021-06-29

**Authors:** Alyssa M. Lanzi, James M. Ellison, Matthew L. Cohen

**Affiliations:** a Department of Communication Sciences & Disorders, University of Delaware, Newark; b ChristianaCare Swank Center for Memory Care and Geriatric Consultation, Wilmington Hospital, DE; c Center for Health Assessment Research and Translation, University of Delaware, Newark

## Abstract

**Purpose:**

Persons with dementia and mild cognitive impairment (MCI) are major consumers of services provided by speech-language pathologists (SLPs). These services include not only direct assessment and treatment of communication and swallowing but also counseling, collaboration, prevention, and wellness. These “counseling+” activities can be especially challenging for SLPs to deliver because of the lack of evidence, as well as the complex nature of Alzheimer’s disease (AD) and other conditions that cause MCI and dementia.

**Method:**

This tutorial is written by a speech-language pathologist, a neuropsychologist, and a geriatric psychiatrist to provide education, resources, and recommendations for SLPs delivering counseling+ activities to patients with MCI and dementia from AD and related disorders.

**Results and Conclusions:**

We describe counseling+ activities across the continuum of care ranging from educating and conducting cognitive screenings with adults experiencing age-related cognitive decline to supporting end-of-life wishes. Because of their expertise in communication, SLPs can provide an array of important leading and supporting services to patients, their family, and other health care professionals on the care team, such as providing patients with appropriate feedback following a cognitive screening and helping caregivers identify the communicative intent of a responsive behavior. The demand for SLP services for patients with MCI and dementia will grow significantly over the next few decades, necessitating more systematic research and clinical evidence in this area.

In a recent survey conducted by the American Speech-Language-Hearing Association (ASHA, 2019), speech-language pathologists (SLPs) reported that 14% of their time working in adult health care settings was spent with persons with dementia (PWDs). This was the second largest reported area of clinical practice (second only to swallowing), indicating that individuals with dementia are major consumers of speech-language pathology cognitive-communication services (ASHA, 2019). Indeed, the need for services for PWD will increase significantly as the “baby boomer” generation progresses through the age band of greatest risk for dementia. The prevalence of dementia due to Alzheimer’s disease (AD) alone is expected to triple in the next 30 years, affecting approximately 15 million U.S. residents and becoming one of the greatest burdens on the health care system (Alzheimer’s Association, 2019).

Despite the large and growing need for dementia-related services, speech-language pathology research and clinical education related to PWDs have been relatively neglected. A recent survey of SLPs working in acute care settings suggests that many clinicians lack confidence and specialized training in the management of cognitive-communication disorders (Morrow et al., 2021). Unfortunately, many graduate programs do not require or offer a stand-alone course in cognitive communication or dementia, despite the fact that the Council on Academic Accreditation in Audiology and Speech-Language Pathology includes cognitive aspects of communication as a necessary learning construct (Morrow et al., 2021). Furthermore, in a recent review of clinical practice research published in ASHA journals over the past 11 years (2008–2018), 46 articles focused on neurodegenerative conditions, in contrast to 108 articles published on the topic of stroke (Roberts et al., 2020). These contrasting figures highlight the mismatch between the amount of research or clinical evidence on speech-language therapy for dementia and the clinical need for such evidence.

ASHA’s Scope of Practice in Speech-Language Pathology (2016) states that, in addition to providing assessment and treatment services, SLPs also perform *counseling*, *prevention and wellness*, and *collaboration* activities. *Counseling* is defined as “interactions related to emotional reactions, thoughts, feelings, and behaviors that result from living with the communication disorder, feeding and swallowing disorder, or related disorders” (ASHA, 2016, p. 9). *Prevention and wellness* is defined as “reducing the incidence of a new disorder or disease, identifying disorders at an early stage, and decreasing the severity or impact of a disability associated with an existing disorder or disease*”* (ASHA, 2016, p. 10). *Collaboration* is defined as “joint communication and shared decision making among all members of the team, including the individual and family, to accomplish improved service delivery and functional outcomes for the individuals served*”* (ASHA, 2016, p. 8). Although ASHA describes these domains separately, they are not mutually exclusive and require some of the same underlying clinical skills, such as reflexive listening, empathy, and patient-centered thinking (Doud et al., 2020). Therefore, for ease of communication, we will call these three domains “counseling+” activities, to serve as a reminder that counseling may include those activities that are traditionally thought of as counseling, plus others that are less obvious.

Unfortunately, many SLPs find these counseling+ activities to be nebulous and lack confidence in their ability to perform them for any clinical population (Phillips & Mendel, 2008). This makes sense given that many SLPs report receiving no training in counseling (Doud et al., 2020; Phillips & Mendel, 2008; Rose et al., 2014). Although research has not been conducted explicitly on SLPs’ counseling+ activities for PWD, we suspect that SLPs feel even less competent and confident providing such services for PWD and their caregivers than for other populations.

To help address some of the issues that arise at the intersection of this complicated and neglected clinical population and the counseling+ activities of practice that many SLPs find vague and uncomfortable, this paper is written by an SLP (author A. M. L.), a neuropsychologist (author M. L. C.), and a geriatric psychiatrist who directs a memory care center (author J. M. E.). Specifically, we aim to discuss the multifaceted role that SLPs have in providing counseling+ activities to patients and caregivers throughout the continuum of care, from typical aging to end of life. We focus mostly on dementia due to AD, but this content may apply to other causes of dementia as well.

## Introduction to Dementia and Mild Cognitive Impairment

*Dementia* is not a medical disease in and of itself, but rather a term used to describe the syndrome of cognitive, functional, and behavioral consequences associated with various diseases. This tutorial focuses on older adults, for whom AD is the most common etiology of dementia (about 60%–80% of cases; Alzheimer’s Association, 2020), but neurocognitive disorders can also be caused by cerebrovascular disease, synucleinopathies such as Lewy body dementia, frontotemporal lobar degeneration, and other pathologies (e.g., James & Bennett, 2019). Instead of the term *dementia*, the 5th edition of the Diagnostic and Statistical Manual of Mental Disorders (DSM-5; American Psychiatric Association, DSM-5 Task Force, 2013) uses the term *major neurocognitive disorder* to describe the same condition—a subjective (i.e., patient-, informant-, or clinician-reported) and objective (i.e., performance-based) decline in cognitive skills from a previous level that overwhelms the person’s ability to compensate and restricts their ability to live independently. One reason the DSM-5 uses the term *major neurocognitive disorder* is that the term *dementia* has become associated with geriatric and progressive causes of dementia such as AD, adding stigma when applied to younger adults with TBI or stroke (American Psychiatric Association, DSM-5 Task Force, 2013, p. 201).

A related condition is *mild cognitive impairment* (MCI or *mild neurocognitive disorder* in the DSM-5), which describes a subjective and objective decline in cognitive skills from a previous level, which does not restrict a person’s ability to live independently, though compensatory behavior is required in order to maintain effective functioning (American Psychiatric Association, 2013). An important point that will be discussed later is that improving a person’s ability to compensate for cognitive vulnerabilities and remain functionally independent by definition staves off a diagnosis of dementia (American Psychiatric Association, DSM-5 Task Force, 2013).

For many people, MCI precedes dementia and so is considered a prodrome. However, it is also a state of clinical uncertainty. Depending on the operational criteria used to define MCI (e.g., cognitive test[s] used, number of cognitive domains assessed, number of low test scores, severity of low test scores), up to 50% of people with MCI may remain stable or in fact improve to an age-typical level (G. E. Smith & Bondi, 2013). For this reason, a diagnosis of MCI should be seen as an opportunity for early intervention rather than a conclusive predictor of dementia. In fact, the World Health Organization estimates as many as 40% of cases of dementia might be prevented or delayed through achievable lifestyle interventions (Livingston et al., 2020).

A few causes of MCI or dementia in older adults are potentially reversible—about 5%–10%, although the estimate is inconsistent in the literature (Clarfield, 2003; Muangpaisan et al., 2012). However, there are no cures for the most common causes: AD, vascular cognitive impairment, Lewy body disease, or frontotemporal lobar degeneration. Individuals living with AD will generally experience a slow worsening of symptoms over time, requiring extensive and comprehensive care for their complex needs from various health professionals, formal caregivers (e.g., certified nursing assistants), and informal caregivers (e.g., family members and friends). Unfortunately, informal caregivers are responsible for the large majority of care. In fact, it is estimated that informal caregivers provide approximately 17.5 billion hours of annual care to PWDs from AD, causing significant physical, economic, and emotional stress (Alzheimer’s Association, 2019; Batsch & Mittelman, 2012; Jolley & Benbow, 2000). Therefore, it is important for SLPs, when possible, to include caregivers when providing counseling+ services to PWDs.

### The Interdisciplinary Dementia Care Team

Although dementia due to AD is a challenging and chronic condition, patients and caregivers can have a good quality of life when provided strong support and adequate care (Batsch & Mittelman, 2012). Person-centered, interdisciplinary care is essential to ensure effective and coordinated management of the disease (Galvin et al., 2014). Once MCI or dementia is diagnosed, the patient’s primary care clinician (PCC) or a geriatric care manager assume management of the patient’s and caregivers’ needs. Depending on the person’s needs and the availability of specialists, the PCC may refer to a psychologist, neuropsychologist, or geriatric psychiatrist for more detailed assessment of cognitive strengths and weaknesses or evaluation/treatment of noncognitive symptoms and other behavioral factors. A neuropsychologist is a clinical psychologist with specialty training in the assessment and treatment of cognitive disorders. A neuropsychological assessment can help improve the accuracy of diagnosis, particularly for complex cases, as well as contribute to treatment planning (Ruchinskas & Cullum, 2018). The geriatric psychiatrist is trained to evaluate the appropriate management of symptoms with techniques that may include behavioral management, pharmacotherapy, and other approaches. The PCC may also refer to a geriatric care manager, often a specialized nurse or social worker, to help navigate social services. This care manager may talk with the PWD and their family about designation of a surrogate decision maker, preparation of an advance directive, and planning regarding financial affairs. Estate planning may require involvement of an attorney with expertise in elder law (Crooks & Geldmacher, 2004; Grand et al., 2011). An occupational therapist (OT) or perhaps registered nurse may assess and improve the person’s ability to live safely at home (e.g., by optimizing lighting, remove tripping hazards, identifying the need for grab bars, etc.; Graff et al., 2006) A physical therapist (PT) may be helpful in maximizing balance, gait, endurance, strength, and flexibility to support independent living (Weber et al., 2011). SLPs contribute expertise in the assessment and optimization of safe swallowing procedures, cognitive rehabilitation, and communication challenges (Swan et al., 2018). *Cognitive rehabilitation* includes a spectrum of activities designed to enhance cognitive weaknesses and/or a person’s ability to compensate for them, typically for people who are community dwelling. Cognitive rehabilitation activities may also be provided by psychologists and OTs (Cicerone et al., 2011).

Each member of the team has unique but also overlapping skill sets, so it is essential that team members communicate effectively, treat one another with respect, avoid “turf wars,” and adapt their services to meet the evolving needs of the patient and caregivers throughout the continuum of the disease (Batsch & Mittelman, 2012; Galvin et al., 2014). We recognize that every PCC coordinates care differently, and each health system and provider have unique practice constraints; however, it is imperative that each provider communicates with other members of the care team to keep them informed, especially because the PWD is even less likely than other patients to facilitate communication among their providers and reconcile miscommunications.

## Counseling+ Roles of the SLP in Dementia Care

**Figure 1.**
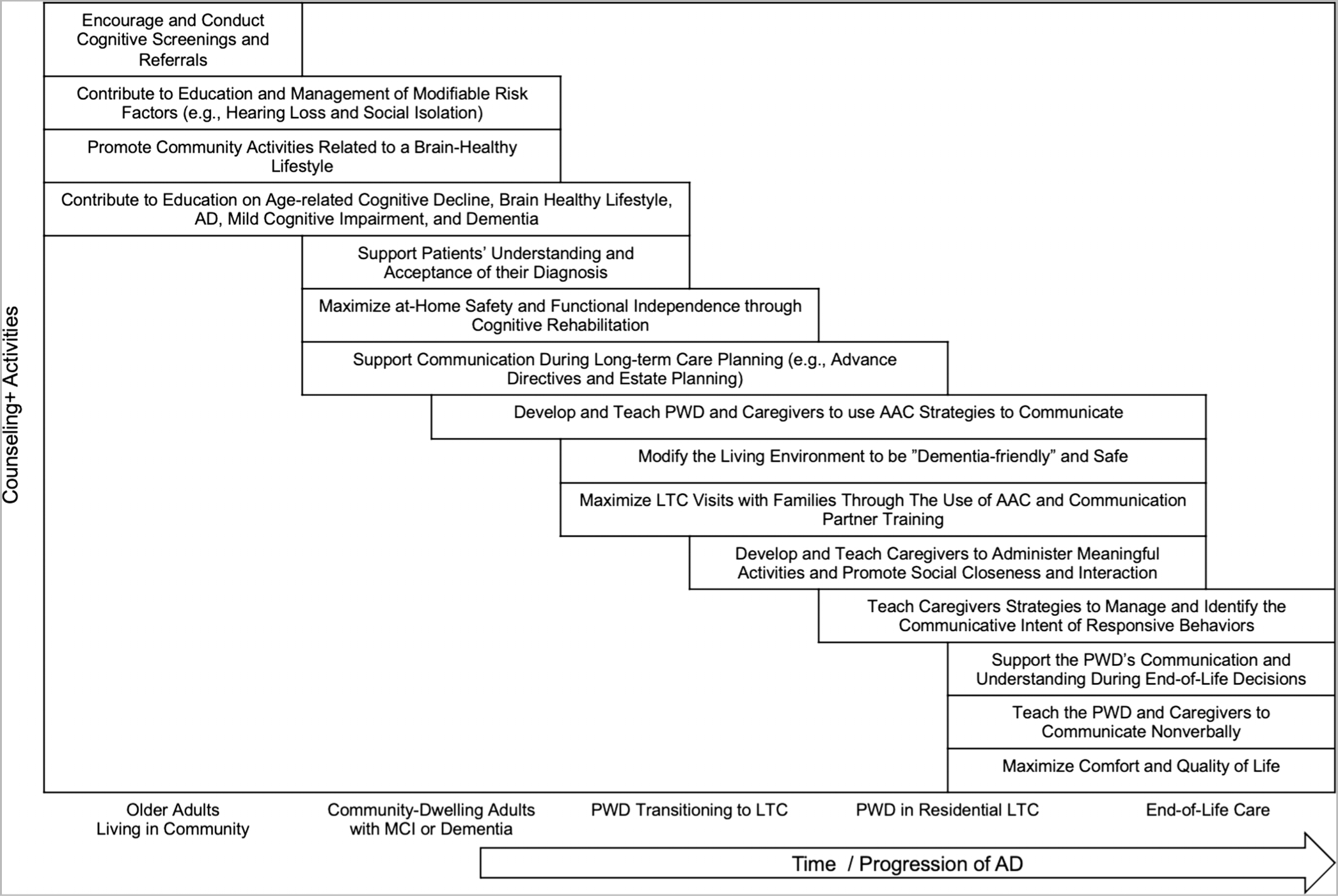
Counseling+ activities delivered by speech-language pathologists throughout the continuum of care for MCI and Dementia. LTC = long-term care; AD = Alzheimer’s disease; AAC = augmentative and alternative communication; PWD = persons with dementia; MCI = mild cognitive impairment.

As illustrated in Figure 1, the remaining sections of this paper will describe the counseling+ activities that SLPs can provide throughout the continuum of care. These services are administered in tandem with typical screening, assessment, and treatment services. Although SLPs may not always be the primary health care professionals doing these counseling+ activities for PWDs, we intend to highlight the important supporting contributions that SLPs can make because these activities rely so heavily on language and communication. Although our focus is on PWDs, we will begin by discussing adults in their 50s and 60s who may be noticing declines in cognition and are concerned about whether those declines are normal. Subsequent sections of this tutorial include content that SLPs should know about MCI and dementia, followed by specific recommendations for counseling+ activities related to that content.

### Counseling+ Activities for Older Adults Living in the Community

There are many misunderstandings about dementia and especially AD (Batsch & Mittelman, 2012; Garand et al., 2009; Jolley & Benbow, 2000). One particularly harmful misunderstanding is that dementia is an expected and inevitable consequence of aging (Cahill et al., 2015). Usage of nonspecific and nonmedical terms like “senility” or “old-timers’ disease” may reflect the confusion that many people have about normal versus pathological cognitive trajectories in aging. Cognitive changes associated with AD and similar conditions are typically insidious (Harada et al., 2013), and it can be difficult to know whether and when to refer to a specialist for consultation. However, a better understanding of typical cognitive aging can help SLPs discern a typical versus atypical experience of cognitive errors and inefficiencies.

Like most other organs, the brain does not operate as efficiently in older age as in younger age. On average, the human brain operates at peak efficiency in the mid-to-late 20s, with efficiency declining slowly but steadily over subsequent decades (Salthouse, 2010). This loss of efficiency is seen on tasks of so-called *fluid cognitive abilities*, which are those required to reason, use information rapidly and flexibly, and problem solve about information that is less familiar or not yet learned (Li et al., 2004; Salthouse, 2010, 2012). Fluid cognitive abilities are those that are assessed by most cognitive tests like attention, processing speed, working memory, episodic memory, and executive functioning. The reader is referred elsewhere for more on the biological and cognitive mechanisms underlying these expected age-related changes, although they are not entirely understood (Heilman & Nadeau, 2019; Park & Schwarz, 2012).

It is common for people of all ages to experience cognitive errors and inefficiencies like occasional word-finding problems, walking into a room and forgetting one’s intention, misplacing keys, forgetting items from a mental list, and so forth. (Cohen et al., 2019). These experiences happen more frequently in advanced age, so it is not necessarily a sign of AD when an older adult is, for example, slow to retrieve an intended word. However, such experiences become more worrisome when they are experienced at a greater frequency or severity than is typical for other people of the same age. It is for this very reason that the normative samples for most neuropsychological tests are stratified for age and other relevant variables like educational attainment. For example, on the 60-item Boston Naming Test (Kaplan et al., 1983), a performance at the 50th percentile for a 56-year-old is 55 or 56 items named correctly out of 60, while a performance at the 50th percentile for a 90-year-old is between 43–48 items named correctly (Ivnik et al., 1996). The distinction between normal age-related word finding challenges and anomia associated with a pathological process can be challenging, so assessments of naming must compare the patient’s performance to a reference sample that accounts for age and education.

At the same time that age is associated with a decline in the brain’s efficiency (fluid cognitive abilities), it is also associated with an increase in knowledge, wisdom, and experience. Crystalized cognitive abilities are those acquired through education, experience, and socialization (Lezak et al., 2004). To illustrate the difference between fluid and crystallized abilities in everyday life, imagine two homeowners preparing for a bad storm, one who is 30 years old and one who is 75 years old. The younger homeowner is more likely to remember a long shopping list without writing it down, scan the aisles of the home improvement store more quickly, read and understand labels and instructions more quickly, and construct equipment more efficiently (i.e., fluid cognitive abilities). However, the older homeowner who has experienced more storms in the same house may make more strategic purchases, know where in the yard to put the generator, better anticipate likely problems, and be wiser about how to allocate their effort (i.e., crystallized cognitive abilities). It is more difficult to quantify these crystallized abilities with performance-based tests; the most common tests of these abilities assess vocabulary knowledge and pronunciation of irregular words. For example, one’s ability to pronounce *sword* and *salmon* correctly are based exclusively on one’s level of education, experience, and socialization.

#### Memory Screening

Although a certain degree of cognitive and functional change is expected with age, some older adults experience a level of decline that exceeds that of their peers and may be indicative of an age-correlated neurological condition, such as AD (Petersen, 2004). Therefore, it is important to screen the cognitive and functional skills of older adults *early* and *often*. In fact, cognitive screening is recommended for Medicare recipients (adults 65 years and older in the United States) during their annual wellness visit (Cordell et al., 2013). The purpose of a cognitive screening is *not* to diagnose, but rather to identify those who would benefit from further evaluation.

Although SLPs may conduct cognitive screenings, it is beyond their scope of practice to diagnose MCI or dementia due to AD. Due to the brevity of most screening tools and natural constraints on health care working environments, it can be challenging to conduct these screenings in a way that does not excessively alarm the patient and to counsel them effectively about next steps. One important step is to ensure that the screening environment is welcoming and comfortable. Many older adults experience anxiety regarding their memory and often fear seeking help from health professionals (Corner & Bond, 2004). We encourage SLPs to start by explaining that the purpose of the screening is not to diagnose but to see whether a diagnostic assessment is needed. It can be helpful to ask at the onset about the patient’s expectations of their performance, because if a referral for a diagnostic assessment is needed, the clinician can connect their recommendation to what the patient already believed, as discussed in the next section.

Because almost no test has perfect diagnostic validity, an SLP performing a cognitive screening should select a test and cutoff score that has a high degree of sensitivity, which means efficient detection of individuals who truly have the condition of interest (“true positives”). However, it is important to know that high sensitivity may be accompanied by lower specificity, which means that some people without a cognitive disorder will also screen positive (“false positives”). The purpose of a follow-up diagnostic exam is then to distinguish the true positives from the false positives. When picking a screening tool and cutoff score, the SLP needs to balance administration time against test reliability and validity. It is also important to know the tool’s sensitivity to MCI and not just dementia. The Mini-Cog (Borson et al., 2003), for example, only takes 5 min or fewer to administer and is relatively sensitive to more severe problems (dementia), but less sensitive to mild problems (Seitz et al., 2018). The Montreal Cognitive Assessment (MoCA; Nasreddine et al., 2005), which takes 10 min to administer and score, is impractical for some settings but is more sensitive to MCI (Lonie et al., 2009). There are over a dozen cognitive screening tools available for SLPs to use for cognitive screenings (Ismail et al., 2010; Yokomizo et al., 2014).

In addition to a performance-based cognitive screening test, it can be useful for the SLP to learn about the patient’s functional skills, for example, by having the patient or an informant complete a questionnaire like the Short Informant Questionnaire for Cognitive Decline in the Elderly (Jorm & Jacomb, 1989) and/or a short interview. It is important to focus on *declines* from previous functioning that are in fact related to cognition and not motor or sensory function (e.g., vision). Including a collateral informant in the screening when possible is valuable because one consequence of cognitive disorders can be restricted insight into one’s own challenges. No one source of information (patient report, informant report, performance-based screening) is entirely without bias, but when considered together, the clinician should have a good sense of whether a further diagnostic assessment is warranted.

The final component of the memory screening is to provide feedback and recommendations. This can be an anxiety-inducing process for both the patient and clinician because of the ubiquitous fear and stigma associated with dementia (Iliffe et al., 2003). Therefore, it is important to remind the patient (and yourself) that the purpose of the memory screening is not to diagnose but to see whether further diagnostic assessment is indicated. A patient should not leave the screening thinking that they do or do not have MCI or dementia; rather, they should leave feeling educated about their performance and resources regarding next steps. If the patient screens negative for a possible cognitive impairment, the main purpose of feedback is to provide some reassurance and encouragement to continue or improve brain-healthy lifestyle choices (see below) and to encourage the patient to talk to their PCC if their concerns persist. Indeed, a negative screen is not necessarily a clean bill of health. False negatives are of course possible, particularly for people in the higher and lower intellectual ranges and for racial minorities (Ruchinskas & Cullum, 2018). Feedback to people who screen negative could be something like “your performance on this test makes me think that your cognition is normal for your age. However, if you or someone close to you thinks that something is wrong, you should still talk to your PCC.” Whenever possible, screening feedback should also be provided in writing for the patient to keep and share with their PCC. The website for the Alzheimer’s Association—which in fact has resources for other causes of dementia in addition to AD—contains several user-friendly resources for educating and counseling older adults on the type of professionals who evaluate thinking and memory, the diagnostic process, and Medicare coverage (Alzheimer’s Association, 2017).

#### Modifiable Risk Factors and a Brain-Healthy Lifestyle

A common misconception is that AD is inevitable for those with a familial history of it. While it is true that patients with a parent, brother, or sister diagnosed with AD are at higher risk of developing the disease, this risk is due to many reasons, including genetic (hereditary), personal, and environmental factors (Alzheimer’s Association, n.d.). Very few people (only 1% or fewer) have genotypes that will inescapably cause them to develop AD (Alzheimer’s Is, n.d.). These are some of the people for whom AD is clinically evident at ages younger than 60. Other people have genotypes that increase but do not guarantee the likelihood of developing AD. The good news is that many risk factors are modifiable or potentially modifiable, and there are lifestyle changes that might delay or prevent the onset of dementia (Livingston et al., 2020).

Unfortunately, the general public’s knowledge of modifiable risk factors for dementia is poor (Cahill et al., 2015). Thus, some of the counseling+ roles of the SLP should be to collaborate with other team members to provide education, assess what is potentially modifiable for that patient, and make strategic recommendations. This is similar to the role that SLPs have in interdisciplinary teams in health care settings for stroke prevention (Miller et al., 2010).

**Figure 2.**
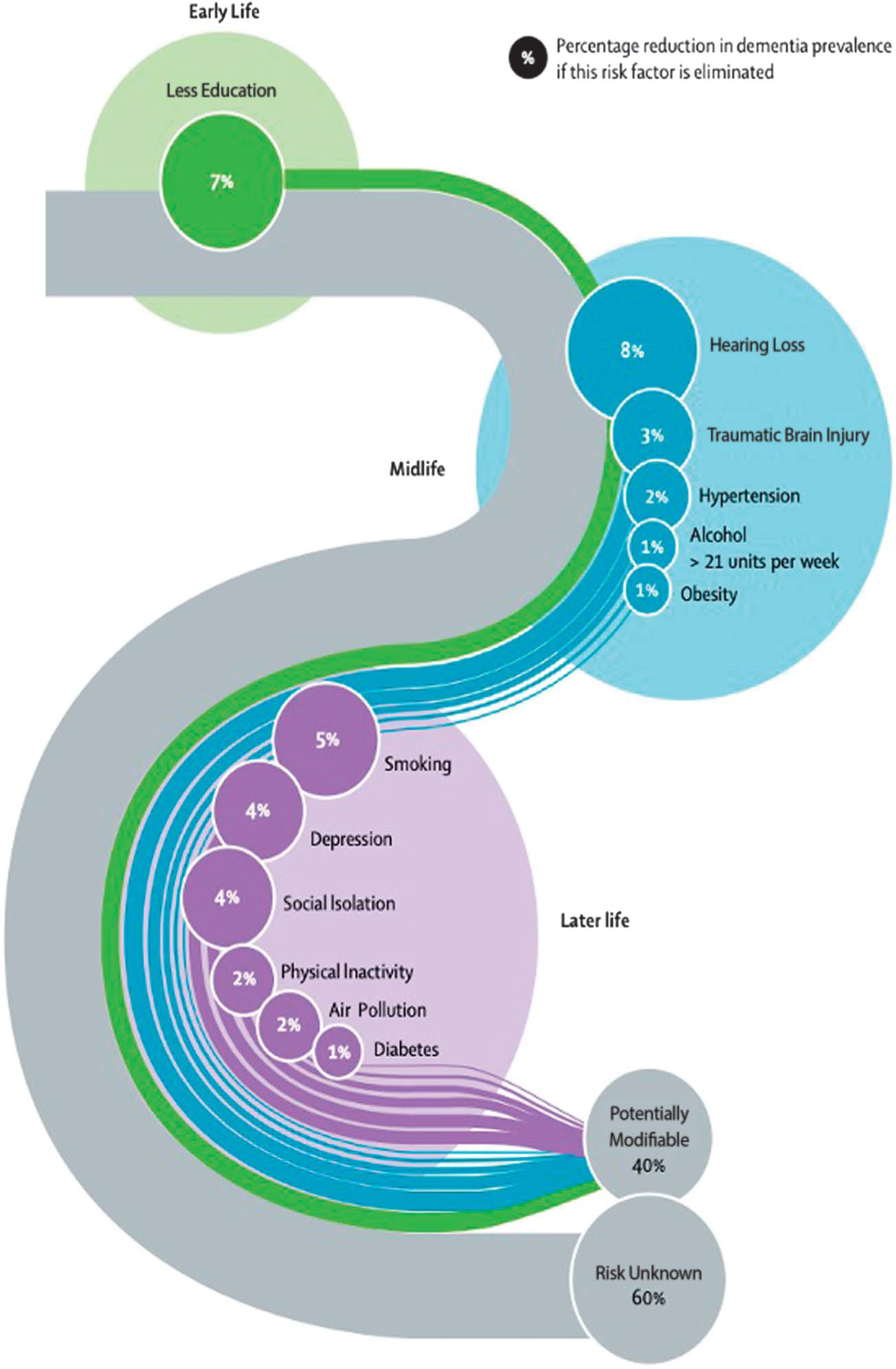
Life-course model for potentially modifiable risk factor for dementia. The figure was originally printed in Livingston et al. (2020) and is reprinted here with permission from the publisher.

Livingston et al. (2020) conducted a literature review and outlined several recommendations for dementia prevention and early intervention. Using a novel life-course model of risk and derived population attributable fractions, they determined that up to 40% of cases of dementia are potentially modifiable. As shown in Figure 2, the researchers specifically identified twelve potentially modifiable risk factors throughout the lifespan: *early life*, (a) education; *midlife*, (b) hearing loss, (c) traumatic brain injury, (d) hypertension, (e) alcohol > 21 units per week, and (f) obesity; and *late life*, (g) smoking, (h) depression, (i) social isolation, (j) physical inactivity, (k) air pollution, and (l) diabetes. Of particular note to SLPs is the importance of hearing loss and social isolation. Encouraging hearing protection in early and midlife, screening for hearing loss, the use of hearing aids when needed, and auditory rehabilitation may help to mitigate the potential risk factor of hearing loss (Liu & Lee, 2019). Furthermore, SLPs can deliver interventions that aim to enhance a patient’s communication skills and engagement in cognitively stimulating activities to help mitigate social isolation in late life (Lanzi et al., 2017). Livingston et al. (2017) also outlined several pharmacological and behavioral interventions that may help, including antihypertensive medications, Mediterranean-style diets, cognitive interventions, exercise, and social engagement.

##### Prevention education.

When talking with older adults in the community and in the memory disorders clinic that he directs, author J. M. E. uses the memorable acronym “DANCERS” to help educate patients about a brain-healthy lifestyle (Livingston et al., 2017; Ngandu et al., 2015). “DANCERS” stands for **D**isease Management, **A**ctivity, **N**utrition, **C**ognitive Stimulation, Social **E**ngagement, **R**elaxation, and Successful **S**leep. It is important to note that not all of these risk factors are equally modifiable *for each patient*. For example, a person with significant dietary or activity limitations may not be able to make significant changes in those areas. Additionally, behavior change of any sort (e.g., weight loss, smoking cessation, exercise) requires significant behavioral support, encouragement, and overcoming of obstacles, so SLPs should collaborate with other professionals (e.g., health psychologists when available) to help the patient achieve the most success.

**D** is for **D**isease Management, which primarily refers to the treatment or management of risk factors, such as, diabetes, hypertension, obesity, smoking, and hearing loss (Livingston et al., 2020). Assessment of current medications, which may increase cognitive difficulties through toxic effects or interactions, is often of value.

**A** is for physical **A**ctivity such as resistance training, aerobic exercise, and tai chi (Kelly et al., 2014). Recent research suggests that even light physical activity has positive benefits (Spartano et al., 2019).

**N** is for **N**utrition, which primarily refers to caloric intake, limiting unhealthy fats, and emulating diets that are associated with reduced risk of cognitive decline. For example, research indicates that Mediterranean-style diets promote healthy aging and may slow cognitive decline for those adults at risk for dementia (Valls-Pedret et al., 2015). This diet consists of olive oil as the main dietary fat with an emphasis on vegetables, fruits, red wine, fish, legumes, nuts, and poultry and a de-emphasis on red meat, butter, cream, and pastries. We direct readers elsewhere to learn more about this diet and to see example meal plans (Romagnolo & Selmin, 2017).

**C** is for **C**ognitive Stimulation, which refers to a set of activities, techniques, or strategies aimed at stimulating a patients’ cognitive skills. Examples of cognitively engaging leisure activities include reading, learning new skills, drawing, and music (Influence of Leisure Activity on the Incidence of Alzheimer’s Disease, n.d.). There is a common belief among older adults that crossword puzzles have particular potency in preserving cognitive skills. To our knowledge, there is no evidence of special value for crossword puzzles. In fact, the best evidence for any leisure activity is still correlational; those who engage in cognitively stimulating leisure activities have a reduced risk of AD, but a strong causal link has not (yet) been established (Stern & Munn, 2010). We recommend to our patients that they pursue activities they find challenging *and enjoyable*; crossword puzzles may be beneficial but are not worth pursuing over other important daily activities or if they are not enjoyable.

A growing body of literature to supports the use of compensatory strategies that help cognitively declining older adults compensate for cognitive losses. It is important to note that these compensatory aids (e.g., using calendar and note-taking systems) are not effective when only passively recommended to older adults (G. Smith & Winegardner, 2020); rather, they require intentional training, for example, through evidence-based compensatory treatment programs, which can be delivered by SLPs (Lanzi & Bourgeois, 2020; Rodakowski et al., 2015).

**E** is for Social **E**ngagement, because loneliness has been identified as a risk factor in cognitive decline (Kuiper et al., 2015). Social engagement refers to both participation in social activities and frequency of social contact with others. It is important for older adults to engage in meaningful and purposeful activities with others (Kuiper et al., 2015). We encourage SLPs to learn more about the activities available to older adults in their communities (e.g., church, senior centers, libraries, Osher Lifelong Learning Institute).

**R** is for **R**elaxation, which may come in the form of mediation, yoga, or muscle relaxation training. These programs are designed to help reduce the negative effects of stress hormones and inflammation and to prevent or help combat depression and anxiety (Klainin-Yobas et al., 2015).

**S** is for Successful **S**leep, which can be promoted through cognitive-behavioral therapy for insomnia (Cassidy-Eagle et al., 2018; McCrae et al., 2020), sleep hygiene, bright light therapy, environmental modifications, medications, or treatments for a primary sleep disorder such as apnea or rapid eye movement behavior disorder (Emamian et al., 2016). One function of deep sleep is to allow the brain a time to clear toxins, including amyloid (one of the proteins that is associated with AD; Ju et al., 2013). Research suggests that a multifactorial approach to sleep interventions may delay the progression of the disease (Landry & Liu-Ambrose, 2014).

##### Community memory screening.

Although in current practice, SLPs primarily conduct cognitive screenings one-on-one with patients who were probably already referred for SLP services, we want to describe an example of an interdisciplinary memory screening model that highlights SLPs as key members of the dementia prevention and wellness team. In 2016, author J. M. E. developed the Memory Ambassadors, a program of trained clinician volunteers committed to helping older adults learn about healthy brain aging and to be screened for early signs of abnormal cognitive decline. The clinician volunteers—and their supervised students—span the disciplines of psychiatry, neuropsychology, SLP, social work, and nursing. Screening events are held in a variety of settings, such as retirement communities and senior centers, including in under resourced communities without good access to specialized care for cognitive disorders. At the start of each screening event, author J. M. E. (geriatric psychiatrist) provides education on dementia and AD and the risk factors and lifestyle modifications that we described earlier in this paper. Following his presentation, the older adult attendees can receive a free wellness screening that assesses nutrition, balance, hearing, and memory. In 2019, the Memory Ambassadors program screened more than 300 adults throughout the state of Delaware. Approximately 30% screened positive and were recommended or directly referred for further diagnostic testing. In fact, if given permission, a memory ambassador can contact that person’s PCC to discuss the findings.

### Diagnosis of MCI and Dementia

A diagnostic evaluation may be triggered when someone screens positive during a Medicare Annual Wellness Visit (which is supposed to include cognitive screening) or other memory screening or if the patient or a significant other seeks the evaluation. MCI and dementia may be diagnosed by physicians (most commonly primary care physicians, psychiatrists, neurologists, or geriatricians), psychologists (most commonly neuropsychologists), or advanced practice clinicians (such as nurse practitioners and physician assistants). The evaluation involves a performance-based assessment of cognitive skills, whether it is a brief assessment done by the physician (e.g., using the MoCA) or a longer assessment done by a neuropsychologist. If there is reason to believe that an abnormal degree of cognitive decline has occurred (or sometimes regardless of performance-based test scores), the consultant will investigate potential causes of that cognitive decline, most commonly with blood tests and structural neuroimaging (Hentschel et al., 2005), looking especially for reversible causes of cognitive decline like medication adverse effects, vitamin deficiencies, hydrocephalus, obstructive sleep apnea, or depression. The diagnostician considers multiple factors when discerning the most likely cause of MCI or dementia, but, for most patients, a state of true certainty about the cause is not achieved. Brain biopsies and advanced neuroimaging are rarely indicated for routine dementia evaluations, so differential diagnoses are based mostly on clinical presentation and limited laboratory evaluation. PWDs who later undergo brain autopsy as part of a research protocol are often shown to have multiple causes (“mixed dementia”), such as small strokes *and* AD pathology (Emrani et al., 2020). AD, being the most common cause of dementia in older adults (about 60%–80% of cases; Alzheimer’s Association, 2020), is often diagnosed in clinical settings by ruling out other conditions with more pathognomonic signs and symptoms. Mood changes like anxiety, depression, and apathy are commonly experienced around the time of diagnosis, perhaps as an early manifestation of the condition, a psychological reaction to it, or both.

#### Recently Diagnosed Patients

There is a lot of variability in how recently diagnosed patients understand, internalize, and respond to their diagnosis of MCI or dementia (Robinson et al., 2011). This variability may stem in part from differences in what patients are told about their condition and its prognosis, what setting in which they are told, whether they have previous experience with MCI or dementia (e.g., caring for a family member), and how much investigation they have done on their own. SLPs should be aware that the person may know little about their condition and they may think that they are simply being referred for treatment of word-finding problems attributing them only to age or without understanding the cause. It is an unfortunate and common occurrence that PWD and their families are often not fully informed about the diagnosis or do not understand its implications (Stokes et al., 2015). It is also possible that PWD may forget what they have been told if it was not written down, and often it is not. This may be compounded by clinicians themselves being uncertain about the diagnosis, having an inadequate understanding about AD or dementia, avoiding unpleasant discussions, or using euphemisms (e.g., saying “memory challenges” and never “Alzheimer’s disease”) with the result that the diagnosis remains unclear (Cody et al., 2002; Connell et al., 2004). There are also some providers who believe that, because there is no cure for AD, early diagnosis is of limited clinical utility and choose not to fully inform the patient of their suspicions (Boise et al., 1999). It has happened in our clinical experience that a patient may be referred to an SLP with an MCI or dementia medical diagnostic code but has little or no understanding or acceptance of their health condition.

Even after a clear conversation about the diagnosis, the patient’s understanding and acceptance of their diagnosis is a process (Aminzadeh et al., 2007), and ideally all members of a treatment team, including SLPs, should play a role in the process. Indeed, a supportive multidisciplinary team has been shown to be associated with patients and caregivers effectively planning for the future, a decrease in caregiver stress, delayed admission to care homes (Brookmeyer et al., 2007; López-Bastida et al., 2009; Rodakowski et al., 2015), and the development of healthy and effective coping strategies that promote successful disease management (Corner & Bond, 2004; Woods, 2001).

Based on our own clinical experiences and on published best practices (Foy et al., 2007; Lee & Weston, 2011), we offer the following recommendations for SLPs if they need to discuss a patient’s diagnosis. To be clear, SLPs should not be the first person to disclose the diagnosis because it is outside of their expertise and scope of practice. Furthermore, there may not need to be a “heavy” discussion if the patient is able to set and make progress towards reasonable and helpful goals. However, there are situations when it is helpful for the patient and clinician to have an explicit conversation about the medical condition and its trajectory, such as when discussing why a patient is only maintaining rather than improving function. During these conversations, SLPs should foster a safe, disarming, supportive, and compassionate environment, for example, by being calm, attentive, and using good reflective listening skills (Zaleta & Carpenter, 2010).

To assess the patient’s level of knowledge, the SLP should ask patients open-ended questions regarding what they already know or suspect about their condition. Hopefully, the SLP will also have thorough notes from other members of the care team to inform the conversation. If not, it is recommended that the SLP proceeds carefully as it is possible that the diagnosis was not disclosed for a specific reason. Ideally, the SLP and the diagnosing clinician should have a correspondence regarding the nature and patient’s understanding of the diagnosis and future roles and responsibilities.

Once the patient is aware of the diagnosis, SLPs should use terminology that is helpful to the patient’s understanding of the condition and pursuit of therapy goals. If the patient forgets or is confused about their condition, it can be helpful to use clear medical terminology like “Alzheimer’s disease.” On the other hand, if the patient understands their condition and is able to set and pursue their goals, describing the symptoms only (“memory challenges,” “word-finding problems”) may be more appropriate to avoid harping on words like “dementia” that are feared and stigmatized.

During goal setting, it may be important to explore what the diagnosis or symptoms mean to the patient. Patients react with different and changing levels of acceptance of the condition, and different and changing postures of response. Some may want to “fight” the condition, and although there is no cure, the patient’s energy might be directed towards “fighting” for a life they want to live despite the condition, such as using compensatory aids and strategies to continue a physically, socially, and mentally active life and meaningful participation in roles and activities. Other patients may feel the full weight of being diagnosed with a terminal illness and withdraw from meaningful activities. A grieving process is of course understandable and normal (Cheston & Bender, 1999), particularly considering how stigmatized dementia remains in our society (Husband, 1999). This grief may be experienced by the patient and their family as sadness, anger, confusion, disbelief, or social withdrawal (Bryden Boden, 2002). Although grief is a normal reaction, a referral to a psychologist or psychiatrist may be warranted to help the patient cope with and prepare for the next chapters of their life, particularly if the patient experiences intense distress or prolonged withdrawal from meaningful relationships and activities. Anxiety, depression, and apathy are also known manifestations of neurological conditions and may benefit from pharmacological treatment.

Although it is not the SLP’s role to provide direct intervention for psychological conditions, it is appropriate and helpful for the SLP to expect and acknowledge those feelings, provide a safe space for the patient to express them—especially when they are relevant to their cognitive-communication goals—and to know when and where to refer the patient for help. The SLP may indirectly help the patient work through grief by collaboratively setting meaningful and person-centered cognitive-communication goals that minimize suffering and maximize strength, function, safety, and meaningful living.

### Staging Dementia

A common question on SLP social media groups and email listservs is about the best way to “stage” dementia, and suggestions from SLPs often include cognitive screening tools like the MoCA (Nasreddine et al., 2005) or clinician-rated tools like the Global Deterioration Scale (Reisberg et al., 1982). In our experience, assigning these values to a PWD does not provide much clinical utility. We encourage the SLP to think about the deeper reason for wanting to “stage” their patient. The motivating question(s) might be:

- How far has this patient declined from their probable baseline?
- Is this person safe to live at home?
- Does this person have the cognitive and linguistic capacity to make health care and/or financial decisions?
- Which community within a nursing home is the best fit for this person?
- What is the right ratio of curative versus palliative care for this person?
- Does this person have the cognitive and linguistic capacity to consent to sexual activity with another resident?

A single metric like the Global Deterioration Scale has almost no clinical utility to answer any of these questions on its own, so we encourage SLPs to think critically about what they really want to know about their patient. Thus, for the purpose of this tutorial, we will organize the subsequent sections according to living environment rather than severity of cognitive impairment. For examples of alternative (although more time consuming) assessment tools, the SLP might consider some of the measures that assess patient preferences for daily living available from preferencebasedliving.com such as the Preferences for Everyday Living Inventory (Van Haitsma, 2013; Van Haitsma et al., 2014).

### Counseling+ Activities for Community-Dwelling Adults With MCI or Dementia

By definition, patients with MCI are able to live independently in the community (or rather, their independence is not significantly restricted by their cognitive limitations). Not all individuals with MCI will progress to dementia but many do—about 10% each year (Bruscoli & Lovestone, 2004; Farias et al., 2009; Mitchell & Shiri-Feshki, 2009). Recent evidence suggests that cognitive rehabilitation may help patients with MCI maintain their independence for a longer period of time (Greenaway et al., 2013; Lanzi & Bourgeois, 2020; Rodakowski et al., 2015). Although extensive discussion on cognitive rehabilitation is outside the scope of this tutorial, we believe that counseling+ activities are often delivered parallel to these evidence-based approaches. Many individuals with dementia also continue to live in their home environment with the assistance and supervision of caregivers. Thus, we suggest the following recommendations to support clinicians in their delivery of counseling+ activities to those patients with MCI or dementia who are still living in the community.

When working with patients who are living in the community, clinicians should support the patient, family, and friends in planning for and coping with possible future declines (e.g., progressive loss of independence and/or transition to supported living). Ideally, someone on the care team should speak with the patient about advance directives, long-term care, health care power of attorney, financial power of attorney, and other estate planning issues. These long-term planning conversations are important for all patients, especially older adults, and particularly those who may have a progressive illness, so that their care progresses according to their wishes. The PCC or social worker will likely take the lead on these conversations, but the SLP can play an important and supportive role. There are several tools that SLPs can use to support patients in effectively communicating their wishes. For example, the Five Wishes planning tool for advance directives (https://fivewishes.org) or the SHARE program: a care planning program for patients and significant others that facilitates early identification of the patient’s core values; development of a care support plan involving the patient, family and friends, and service providers; and activities for stress management (Orsulic-Jeras et al., 2019).

Clinicians can also incorporate long-term planning into therapeutic goals. Because the patient is living in the community, goals should focus on maximizing safety at home and promoting everyday functioning. Goals may also focus on developing augmentative and alternative communication (AAC) strategies, such as Memory Books (Bourgeois, 2007). Developing memory aids early may help ease the concerns of those patients who are anxious about their memory and communication in the future. Caregivers (e.g., family members and friends) may also benefit from formal dementia-based caregiver training programs to support preparedness and long-term care (Pleasant et al., 2020). SLPs can also help by providing communication partner training (E. R. Smith et al., 2011).

### Counseling+ Activities During the Transition to Residential Long-Term Care

Noncognitive behavioral symptoms like depression, apathy, hallucinations, and delusions are often difficult and distressing for family caregivers and may prompt the moving of their loved one to a long-term care home (e.g., assisted living facility). This transition can be challenging for many reasons; however, SLPs can play a supportive role and work with other team members to facilitate a positive transition, for example, by collaborating with a social worker to help the PWD express their input for a future living environment. Upon admission, Medicare or Medicaid-funded care homes are mandated to conduct a standardized assessment of each resident’s functional capabilities and health needs (Centers for Medicare & Medicaid Services, 2012). During this assessment, SLPs can assist the PWD in communicating their preferences for leisure and cognitively stimulating activities using evidence-based visual supports (Burshnic & Bourgeois, 2020). SLPs can also work with the OT, PT, or registered nurse to assess safety and help the caregiver modify the new living environment to be “dementia friendly” using the recommendations discussed below.

The transition from community to supported living can be a stressful time for not only the patient but also the family and friends (Grant et al., 2002). Furthermore, the family and friends may encounter new challenges as they adjust to visiting, monitoring, and advocating for their loved one’s health care (Gaugler, 2005). SLPs can teach family and friends effective communication strategies—using principles of multimodal communication, AAC, and communication partner training—and how to deliver person-centered activities—using principles of Montessori for adults—to ensure that their time spent visiting their loved one is meaningful and enjoyable (E. R. Smith et al., 2011). SLPs can also support the PWD’s receptive and expressive language skills during routine video or telephone calls to improve social communication with family members and friends.

### Counseling+ Activities for PWD During Residential Long-Term Care

In contrast to the cognitive and language skills of individuals with poststroke aphasia or traumatic brain injury, the cognitive and language skills of PWD due to AD or other geriatric neurodegenerative diseases are expected to decline over time despite even the most efficacious interventions. This requires care team members to work with PWD, their family and friends, and formal caregivers to provide rehabilitation to improve and maximize function as much as possible. When rehabilitative therapy is not appropriate, SLPs can design functional maintenance programs for PWD to ensure safety, provide caregiver training, maintain functional capacity, and improve quality of life (Centers for Medicare & Medicaid Services, n.d.). The subsequent sections of this paper provide recommendations and sources for SLPs to incorporate counseling+ activities into their care of patients in residential long-term care settings.

The social environment of PWDs often narrows because of feelings of embarrassment or shame, as well as stigma and discrimination (Garand et al., 2009). Unfortunately, this stigma and discrimination may lead to isolation and negatively impact a PWD’s communication and social skills. SLPs can design a patient-centered group activity to help preserve communication skills, like something related to past interests, hobbies, career, roles, and cultural values of the PWD and focuses on what the patient with dementia *can* do instead of what they *can*’*t* do. To illustrate this concept, imagine that an SLP is working with a Barbara, a PWD in a skilled nursing facility who used to participate in a weekly book club at her local library. The SLP decided to design a maintenance plan that implements the Question Asking Reading procedure during a book club activity (Judge et al., 2000). This activity was chosen for Barbara because book club was an enjoyable pastime of hers and her reading skills are within functional limits. As part of this activity, the PWD will participate in a small-group conversation about a book, on a topic of interest, using external memory aids and visual cue cards. The SLP will teach formal caregivers how to lead this activity, how to support the engagement and communication skills of Barbara, and how to increase the social interaction between group members.

To support care team members in developing a meaningful and safe environment for the PWD, we suggest incorporating principles of Montessori for Dementia and Aging, such as freedom, structure and choice, nature, atmosphere, and specialized materials (Douglas et al., 2018; Montessori for Dementia and Ageing, n.d.). This environment should incorporate multimodal cues (e.g., auditory, visual, olfactory, and tactile) and include items that are meaningful to the PWD, such as personal photographs, old furniture, or personal awards. Within the environment, the SLPs can teach caregivers to develop AAC supports, such as visual cues and signage that gain the PWD’s attention and that promote engagement in a specific task. For example, there could be an area of the common room with different artificial flowers and vases along with a sign that reads, “Please arrange the flowers in a vase.” We also suggest developing visual cues and signage to promote safety and personal hygiene during activities of daily living. For example, there can be a sign with written and visual support describing simple steps to apply hand sanitizer. We direct readers elsewhere to see examples of visual cues and signage for PWD (Dementiability Enterprises Inc., n.d.).

#### Responsive Behaviors

In the later phases of their condition, many PWDs will behave in ways that are disruptive and problematic for caregivers and other residents, such as grabbing onto others, wandering, yelling, hitting, kicking, asking repetitive questions, making loud noises, and engaging in inappropriate sexual behavior. Yous et al. (2019), among others (Dupuis et al., 2012), describe these as “responsive behaviors” because that term “… acknowledge[s] the multiple layers that exist underneath a behavior and the various behaviors that can be used by PWDs to respond to a situation or environment” (2019, p. 2). SLPs may help the care team try to understand the communicative intent of a behavior, if there is one, and work to find a more constructive way for the PWD and caregiver to communicate, such as using AAC and nonverbal communication. For example, a visual schedule can help promote a daily routine with meaningful activities for PWD who exhibit wandering and exit-seeking behaviors. We direct readers elsewhere for additional resources and examples of managing responsive behaviors in PWD (Responsive Behaviours in Dementia—Mount Sinai Hospital—Toronto., n.d.).

### Counseling+ Activities for PWD During End-of-Life Care

As patients progress to the advanced stage of any terminal disease, prognostic markers (e.g., functional dependency, increased hospitalizations, and weight loss) are typically used to predict a life expectancy of less than 6 months and trigger a referral to hospice. Unfortunately, evidence suggests that the use of these prognostic markers for PWD are not reliable at predicting life expectancy (Schonwetter et al., 2003). Furthermore, the cognitive, linguistic, and noncognitive behavioral symptoms experienced by PWD pose barriers to their decision making and comfort during end-of-life care (Sachs et al., 2004). Therefore, the dementia care team plays a major role in assessing, identifying, and managing end-of-life symptoms and decisions. Based on the literature and our experience, we suggest the following recommendations to incorporate counseling+ activities into end-of-life care for PWD.

There are many health care decisions that need to be made during end-of-life care for PWD, such as resuscitation, invasive medical procedures, drug treatment, hospitalizations, and feeding tube placement. SLPs play a primary role in supporting dysphagia decision making, and we direct readers elsewhere to learn more about that area of practice (P. A. Smith, 2020). SLPs can also work with other team members to help the PWD and their family effectively communicate and understand additional medical decisions. Recent evidence suggests that incorporating visual aids into the end-of-life decision-making process enhances PWDs’ understanding, reasoning, and appreciation (Chang & Bourgeois, 2020). Thus, we recommend using AAC strategies to support the communication of PWD and also support the PWD’s understanding of the decision even if the power of attorney will ultimately make the decision.

Coping with end-of-life decisions can be especially challenging for family and friends. Care team members can help the PWD and informal caregivers anticipate these decisions early and revisit the advance care planning decisions that were made earlier in the PWD’s care. It is important that the end-of-life wishes that were communicated by the patient are honored and that families receive support from counselors on how to cope with these decisions. Palliative care, for example, offers counseling and additional support services to family members. We direct readers elsewhere to learn more about palliative care for dementia (Hughes et al., 2007; Murphy et al., 2016).

During end-of-life care, it is also important to maximize the comfort and quality of life for both the PWD and caregivers. We suggest that members of the care team use an evidence-based “high touch” protocol (Simard & Volicer, 2014) to maximize the comfort of PWD. For example, the Namaste Care program (Simard & Volicer, 2014) emphasizes physical touch as a medium of communication and the dual process of both receiving and initiating touch. SLPs can educate caregivers on recognizing forms of nonverbal communication of different emotions, such as distress or happiness. SLPs can also teach caregivers effective examples of nonverbal communication to maintain interpersonal closeness.

## Conclusions

People with MCI and dementia are major consumers of clinical services provided by SLPs, and the demand for these services will increase significantly as conditions like AD increasingly affect the “baby boomer” generation. In addition to providing direct assessment and treatment services, it is within the SLP’s scope of practice to engage in counseling, collaboration, and prevention and wellness activities, which we have called “counseling+” activities. To best meet the needs of PWD and MCI, counseling+ activities should be interdisciplinary and person centered. Indeed, because of their expertise in communication, SLPs can provide important supporting services to other members of the care team to help enhance the lives of patients and their families. This important role starts with prevention and wellness for community-dwelling older adults and progresses through end-of-life care for PWD. We have provided initial education and recommendations for SLPs to use when working with this clinical population. However, significantly more research is needed in this area to support SLPs’ clinical practice and to enhance the quality of life of patients.
